# Exposure to Sub-lethal 2,4-Dichlorophenoxyacetic Acid Arrests Cell Division and Alters Cell Surface Properties in *Escherichia coli*

**DOI:** 10.3389/fmicb.2018.00044

**Published:** 2018-02-01

**Authors:** Supriya V. Bhat, Belma Kamencic, André Körnig, Zinnat Shahina, Tanya E. S. Dahms

**Affiliations:** ^1^Department of Chemistry and Biochemistry, University of Regina, Regina, SK, Canada; ^2^JPK Instruments AG, Berlin, Germany

**Keywords:** 2,4-dichlorophenoxyacetic acid, cell division, correlated atomic force – laser scanning confocal microscopy (AFM-LSCM), DNA damage, FtsA, FtsZ, membrane potential, SOS response

## Abstract

*Escherichia coli* is a robust, easily adaptable and culturable bacterium *in vitro*, and a model bacterium for studying the impact of xenobiotics in the environment. We have used correlative atomic force – laser scanning confocal microscopy (AFM-LSCM) to characterize the mechanisms of cellular response to the herbicide 2,4-dichlorophenoxyacetic acid (2,4-D). One of the most extensively used herbicides world-wide, 2,4-D is known to cause hazardous effects in diverse non-target organisms. Sub-lethal concentrations of 2,4-D caused DNA damage in *E. coli* WM1074 during short exposure periods which increased significantly over time. In response to 2,4-D, FtsZ and FtsA relocalized within seconds, coinciding with the complete inhibition of cell septation and cell elongation. Exposure to 2,4-D also resulted in increased activation of the SOS response. Changes to cell division were accompanied by concomitant changes to surface roughness, elasticity and adhesion in a time-dependent manner. This is the first study describing the mechanistic details of 2,4-D at sub-lethal levels in bacteria. Our study suggests that 2,4-D arrests *E. coli* cell division within seconds after exposure by disrupting the divisome complex, facilitated by dissipation of membrane potential. Over longer exposures, 2,4-D causes filamentation as a result of an SOS response to oxidative stress induced DNA damage.

## Introduction

A global crisis is emerging in which an increase in food demand which has led to the significant use and development of pesticides. Efforts have been increasingly made to develop more potent chemicals to target resistant agricultural weeds. Since the early 1940s, the herbicide 2,4-dichlorophenoxyacetic acid has been one of the most commonly used pesticides in Canada and world-wide to target broad-leaf weeds. This compound persists with a half- life of 10–200 days in the environment and is known to have undesired effects on diverse species in the food chain, from mammals to soil bacteria (Boivin et al., 2005; Chinalia et al., 2007). Environmentally relevant exposure levels have been determined to be 5 mg kg^-1^, however, bacteria are exposed to a wide-range of pesticide concentrations depending on a number of factors such as soil type, moisture content, amount of organic matter and the presence of degrading bacteria (Zabaloy et al., 2010). This herbicide is known to have significant non-target effects and its complete mode of action is not clearly known, even in target species. The herbicide is known to act through a combination of hormonal, oxidative stress and disruptive cell division mechanisms in target plants (Pazmino et al., 2012). It is known to cause membrane defects, disrupt fatty acid biosynthesis, lipid peroxidation, protein synthesis and induce oxidative stress in bacteria (Chinalia et al., 2007; Pazmino et al., 2014; Bhat et al., 2015a). Despite the significant application of this herbicide, its specific effects on non-target species are unknown.

*Escherichia coli* is a robust, easily adaptable and culturable bacterium *in vitro*, making it an excellent model for studying bacterial response mechanisms to xenobiotic exposure. *E. coli* has been used to characterize the impact of several antimicrobial compounds including peptides, antibiotics, pesticides, and other xenobiotics (Guven et al., 2005; Ruiz and Silhavy, 2005; Asghar et al., 2006). Survival of this organism in diverse hostile environments comes from its ability to persist and reproduce (Touchon et al., 2009). Morphological change, often a survival advantage, is one of the key adaptation mechanisms exhibited by bacteria under stressful conditions. In particular, *E. coli* exhibits a filamentous phenotype during host invasion (Henry et al., 2005; Justice et al., 2006), temperature (Ricard and Hirota, 1973), oxidative (Bhat et al., 2015b), and xenobiotic (Boberek et al., 2010; Bhat et al., 2015b) stress. However, it is unclear which molecular mechanisms underlie the filamentation process and whether or not they are unique to particular types of stress.

Cell division in *E. coli* is tightly regulated and it is initiated by the assembly of the divisome complex at the mid-cell septum. FtsZ forms the Z-ring scaffold with other colocalizing partners, many of which are structurally and functionally characterized (Adams and Errington, 2009; Egan and Vollmer, 2013). FtsZ is a GTP-dependent tubulin homolog, and its action is tightly regulated by a number of intracellular and environmental factors. FtsA colocalizes with FtsZ forming actin-like protofilaments, anchoring FtsZ to the inner membrane and helping to recruit downstream proteins to further stabilize the complex. There are several factors known to influence Z-ring formation at the mid-cell, including the action of MinCDE proteins and nucleoid occlusion mediated by SlmA (Monahan et al., 2014). SulA, a product of the SOS response induced following DNA damage during oxidative stress, is also known to inhibit FtsZ polymerization by binding its C-terminus and possibly inhibiting the GTPase activity necessary for its polymerization (Cordell et al., 2003). In response to stress, SulA binds to FtsZ, disassembling existing Z-rings and preventing the assembly of new rings, thereby blocking cell division and preventing damaged DNA from passing to daughter cells (Adams and Errington, 2009).

The divisome is a membrane anchored complex of several proteins which drives the formation of mid-cell constriction. The polymerization of FtsZ, FtsA and recruitment of several other downstream proteins are energy consuming processes, so it is not surprising that the divisome complex is anchored to the membrane, the site of ATP biosynthesis (Loose et al., 2008). ATP synthesis requires maintenance of the proton motive force (pmf), which generates the membrane potential (Δψ) as a result of a proton gradient across the membrane. The proton electrochemical gradient is reflected in Δψ and ΔpH, and a change in one is compensated by the other so that a constant electrochemical gradient is maintained (Zilberstein et al., 1984). A shift in extracellular pH arrests cell division in *E. coli*, affects DNA synthesis and results in long filaments, indicating that cell division is a pH sensitive process (Zilberstein et al., 1984). Dissipation of Δψ directly affects the localization of cell division proteins and causes disassembly of the divisome complex (Strahl and Hamoen, 2010). Recently it has been shown that ionophores such as indole arrest cell division in *E. coli* in a similar fashion (Chimerel et al., 2012).

We previously showed that sub-lethal levels of 2,4-D cause oxidative stress and induce a filamentous phenotype in *E. coli* (Bhat et al., 2015b), leading to the hypothesis that 2,4-D affects components of cell division. Here we provide evidence for the impact of 2,4-D on cell division proteins, DNA damage and simultaneous temporal changes to surface ultrastructure and physical properties. Based on cytological evidence we propose that 2,4-D alters membrane potential, immediately impacting FtsZ, FtsA localization and disrupting cell division, induces oxidative DNA damage and initiates the SOS response, ultimately leading to the filamentous phenotype at longer exposure times.

## Materials and Methods

A commercial formulation of 2,4-D amine salt [w/w % 84.21 2,4-D, 0.5 Triton-X-100, 1.5 EDTA, 1.41 of 60% dimethylamine aqueous solution, and 12.38 of soft water; analysis by Interprovincial Cooperative Limited (Agri Products Department, Winnipeg, Canada)] was purchased from Viterra, Regina and stored in the fume hood at room temperature. HPLC analysis showed the 2,4-D formulation to be stable after 24 h in an aqueous environment (Bhat et al., 2015b).

All other chemicals were analytical grade and purchased from Sigma–Aldrich unless otherwise noted. Water used for media and sample preparation was deionized (18 MΩ, Barnstead Nanopure, Thermo Scientific).

### Strains and Growth Conditions

The *E. coli* strains used in this study, a kind gift from Dr. William Margolin (Geissler et al., 2007), are listed in **Table 1**. A *sulA*p–GFP reporter strain regulated by DNA damage that was introduced into the wild-type WM1074 (WM1074+*sulAp–gfp)* chromosome served as a reporter of the SOS response (Geissler et al., 2007) [original source; (McCool et al., 2004)]. WM2026, WM1074, and WM2739 were grown on Luria-Bertani (LB) broth at 32°C and WM2760 was grown under the same conditions with the addition of 100 μg/mL ampicillin. WM2026 and WM2760 were induced with 40 and 10 μg/mL of IPTG, respectively, 2 h prior to harvest. An overnight culture was used as a stock for inoculating into all the test samples. A formula control, consisting of all formulation ingredients except 2,4-D, and sample controls containing deionized water in place of formulation, were tested in parallel.

**Table 1:** Descriptions of *Escherichia coli* strains used in the current study.

Strain	Description	Source
WM1074 (parent)	Wild type strain, derivative of MG1655 lacU169	Geissler et al., 2007
WM2026 (FtsZ-GFP)	WM1074 + stable chromosomal fusion FtsZ-GFP	Geissler et al., 2007
WM2760 (GFP-FtsA)	WM1074 containing pWM2760 (Ptrc-gfp-ftsA, pBR322 derivative)	Geissler et al., 2007
WM2739 (SulAp-GFP)	WM1074 + stable chromosomal fusion SulAp-GFP	Geissler et al., 2007

The MIC of 2,4-D and the changes to cell length during 2,4-D exposure for *E. coli* WM1074 were determined as previously described (Bhat et al., 2015b). Growth curves for 1 mM and 4 mM 2,4-D were constructed after 30 h incubation in 96 well plates, with the OD_600_ (optical density at 600 nm) measured every 30 min.

### DNA Damage Assay

The extent of 2,4-D induced DNA fragmentation was tested using the agar diffusion method (Fernandez et al., 2008). *E. coli* WM1074 cells grown for 3–4 h were exposed to 0–4 mM 2,4-D (5, 10, 30, and 60 s, 3 h and overnight), H_2_O_2_ (6 h and overnight) and 50°C (2 h). The culture was diluted to approximately 0.1 OD_600_ and 25 μL was mixed with 60 μL of 0.1% molten agarose at 37°C and vortexed thoroughly. A 20 μL aliquot of this mixture was then spotted onto slides pre-coated with agarose (pre-coating involves coating a clean, grease-free glass slide with 0.1% agarose and drying in an oven at 70°C for 2 h) and a coverslip was carefully placed on the sample to prevent air bubbles in the gel. The slide was incubated at 4°C for 10 min allowing the gel to solidify. The coverslip was removed carefully and the slides were incubated in lysis buffer (2% sodium dodecyl sulfate, 0.05 M EDTA, and 0.1 M dithiothreitol, pH 11.5.) at 37°C for 5 min. All samples were submerged in lysis solution in the same tray to avoid bias in treatment. The lysis buffer was removed and the slides carefully washed, without tilting, in deionized water (5×) and dried in ethanol (70, 95, and 100%, 3 min each at -20°C). The slides were then dried in an oven under vacuum overnight and stained with SYBR gold (Life Technologies) for 5 min, washed and mounted in TBE buffer with a 18 × 18 mm Zeiss precision coverslip and imaged by epifluorescence microscopy (Ex: 497 nm, Em: 537 nm, Zeiss Axio Observer Z1). Exposure to elevated temperature and H_2_O_2_ served as positive controls. The degree of DNA damage was determined by measuring the distance from the cell periphery to the edge of the halo around each cell, indicating DNA spreading. Images were processed to maximize contrast and remove background noise (Zeiss Zen software) and the diameter of DNA halos surrounding 100 cells for each sample were measured using ImageJ. Differences in samples were assessed using an unpaired *t*-test in GraphPad Prism 5.

### Membrane Depolarization Assay

To determine the effects of 2,4-D on membrane potential, a membrane depolarization assay was used as described in Te Winkel et al. (2016). Briefly, exponentially growing *E. coli* WM2026 cultures (O.D 0.2–0.3) were exposed to 0, 1 mM and 4 mM 2,4-D in a 96 well plate in LB media. The dye 3,3^′^-diethylthiadicarbocyanine iodide [DisC2(3)] dissolved in DMSO was added 5 min before the addition of test compounds to a final concentration of 2 μM, with the final concentration of DMSO kept at 1%. Valinomycin at 30 μM was used as a positive control. To examine the possibility of DisC2(3) dye reacting with 2,4-D, wells with water, 4 mM 2,4-D and dye were tested simultaneously. Changes to fluorescence intensities were measured on a microplate reader (BioTek-Synergy) equipped with 560 nm excitation and 580 nm emission filters. The measurements were taken for 60 min after the addition of test compounds.

Immediately following 2,4-D exposure, 10 μL of the sample was placed on a microscope slide, covered with a glass coverslip and examined by LSCM (560/590 nm).

### Epifluorescence Microscopy

Samples from the DNA damage assay (above) and the *E. coli* WM2026 strain exposed to 4 mM 2,4-D after 3 h and overnight exposure were imaged (Ex: 488 nm, Em: 509 nm) on a Zeiss Axio Observer Z1 inverted wide-field fluorescence microscope to determine DNA damage, and the Z-ring and nucleoid positioning, respectively. The cells were stained with DAPI (100 μg/mL, Ex: 358 nm, Em: 461 nm) and mounted in 0.01 M PBS and imaged.

Similarly, *E. coli* WM2739 was imaged to quantify the oxidative stress-induced SOS response. Changes to SulAp-GFP intensity were measured using the ZEN software (Blue edition, 2.1 lite) intensity measurement function. Intensity values collected for each of 100 cells were corrected for background, and statistical analysis conducted with an unpaired *t*-test using GraphPad 5.

### Live Laser Scanning Confocal Microscopy (LSCM)

Polystyrene petri dishes with an 18 mm circular hole cut into the bottom were sealed with a Zeiss high precision coverslip placed at the bottom using epoxy resin. The coverslip was pre-cleaned (Bhat et al., 2015b) and then coated with Cell-Tak (Corning) using a slightly modified method (Louise Meyer et al., 2010). A 30 μL aliquot of freshly prepared Cell-Tak solution (145 μL of pH 8 NaHCO_3_ buffer, 5 μL of 1 mM NaOH and 5 μL of Cell-Tak) was spread (1 sq. cm) onto coverslips, incubated (RT, 30 min), rinsed gently with ultrapure water and air dried. The coated coverslips were stored for up to 2 weeks at room temperature.

Approximately 500 μL of the culture was added to the coverslip and incubated (32°C, minimum of 30 min) in the dark. The sample was rinsed with LB diluted 1:1 with PBS (0.01 M, pH 7) and mounted with 500 μL of the same solution for imaging using the 63× oil immersion objective on the LSCM. The PBS and media were filtered (0.2 μm) and maintained at 32°C prior to sample preparation. The petri dish with sample was placed in a heated holder maintained at 32°C. Time lapse images were collected before the addition of 2,4-D, and 2,4-D (0.01–4 mM) was added in real-time during imaging, with images collected for up to 6 h post treatment. Formula solution, not containing 2,4-D, was added for imaging control samples.

To determine the effect of oxidative stress on the localization of FtsZ and FtsA, known ROS inducers – paraquat and H_2_O_2_ were added in real-time during imaging at various concentrations (0.01–20 mM). Similarly, the ionophores vali-nomycin (10–30 μM) and nigericin (5–10 μM) dissolved in DMSO were tested for their effects on the localization of the cell division proteins based on their ability to alter membrane potential. Controls contained the same volume of DMSO only.

### Integrated Atomic Force-Laser Scanning Confocal Microscopy

Our AFM-LSCM setup consists of the Nano Wizard AFM (JPK, Germany) placed on an inverted LSCM 780 equipped with 34 channels of highly sensitive GaAsP detectors, steady state excitation lasers (458, 488, 514, 543, and 594 nm) and a Ti:Sapphire tunable femtosecond pulsed IR laser. The LSCM stage was replaced with the custom designed AFM stage purchased from JPK and the sample mounted in a manner similar to that described above. A camera was mounted onto the front port of the confocal microscope base and connected to the AFM-ECU to produce a low resolution (20×) DIC image of the sample to allow alignment of the laser on the AFM tip. The sample was subsequently viewed using a 63× oil immersion objective, with the focal plane close to the center of the cell. A suitable actively dividing (FtsZ-GFP ring at the mid cell) single cell, which appeared immobile by DIC and confocal, was chosen for imaging. The JPK AFM software optical calibration tool was used to precisely position the AFM tip in relation to the cell for generating optical overlay. A force constant calibration of the AFM cantilevers (Nanoscience, model no: HYDRA4V-100N), having a low nominal spring constant (*k* = 0.08 N/m and calibrated *k* = 0.05 ± 0.03 N/m in imaging media), was followed by a low resolution force map used to evaluate the integrity of the chosen cell. The height image from the force map was used to choose an appropriate area for collecting the QI^TM^ (quantitative imaging) image. Parameters such as set point, Z-length, and approach/retract speed were adjusted for live QI^TM^ to reduce noise. Since AFM-QI and LSCM images are collected over different time scales, LSCM images were collected before and immediately after the completion of QI^TM^ images to demonstrate simultaneous change. Approximately six images collected on different samples and days, at different time points, each with 16834 force curves, were used for processing images before 2,4-D treatment and three images collected on three separate samples and days per time point were used for processing time lapse images during 2,4-D treatment. The AFM and LSCM images taken approximately at the same time were overlaid using Photoshop 11. LSCM images were processed for contrast and digitally enlarged to fit the AFM height image.

QI^TM^ force curves obtained at each pixel on a 128 × 128 image were corrected (JPK software) for the cantilever force constant and baseline tip-sample separation. The adhesion was determined using the distance between the lowest point and baseline of the retract curve. Young’s modulus was determined using a Hertzian fit, which is an estimate of cell envelope elasticity (JPK software). Surface roughness was measured at the mid-point of the cell using the QI^TM^ height images as previously described (Bhat et al., 2015b). All the force curves in the image were batch processed using a 200 nm × 200 nm square in the middle of the cell. Histogram data was exported from the JPK software for statistical analysis and plotting. All data were statistically analyzed using unpaired student’s *t*-test and one-way ANOVA (GraphPad Prism 5).

## Results

The minimum inhibitory concentrations (MIC) of 2,4-D for all strains used in this study were determined to be 6 mM, so 4 mM was used as the highest sub-lethal concentration that produced consistent and sufficient cell growth for microscopy and biochemical assays. The growth curve showed a significant reduction in growth with 4 mM 2,4-D exposure compared to 1 mM and controls (**Supplementary Figure S1**). Growth appeared slightly slower between 12 and 15 h for samples exposed to 1 mM 2,4-D, but the growth after 15 h appeared similar to controls.

### 2,4-D Causes DNA Damage during Short and Long Time Exposures

**FIGURE 1:**
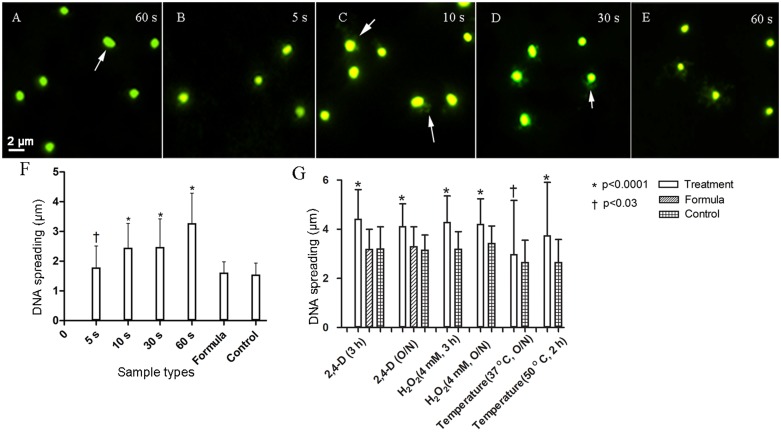
Fluorescence images (SYBR gold 497/537 nm) showing immediate DNA damage and plots showing increased DNA fragmentation after short **(F)** and long **(G)** exposure to 1 mM and 4 mM 2,4-D, respectively. *Escherichia coli* exposed to 1 mM 2,4-D showed increased DNA damage measured as a function of increased DNA spreading after 5 s **(B)**, increasing further after 10 **(C)**, 30 **(D)**, and 60 s **(E)** compared to the representative formula control **(A)**. Arrows highlight halos around the cells. Formula sample contained all the ingredients of the formulation (refer to section “Materials and Methods”) without 2,4-D and the sample control contained deionized water. Cells also showed significantly increased DNA damage after longer exposures to 4 mM 2,4-D (*n* = 100). H_2_O_2_ and high temperature were used as positive controls. Error bars indicate standard deviation.

The extent of DNA damage was determined by monitoring fluorescently labeled cells for DNA spreading as a function of DNA damage in *E. coli* WM1074. These changes were examined during short and long time exposures by measuring the DNA halo around single cells, a direct indication of fragmented DNA. After a 5 s exposure to 1 mM 2,4-D, there was a statistically significant (*p* < 0.03) increase in DNA damage, which further increased (*p* < 0.0001) after 10, 30, and 60 s exposures compared to controls after 60 s exposure (**Figure 1** and **Table 2**). The DNA damage assay after 3 h and overnight exposure to 4 mM 2,4-D showed significantly increased DNA fragmentation (*p* < 0.0001, *n* = 100) compared to formula and sample controls (**Figure 1** and **Table 2**). Positive controls, in which oxidative stress had been induced for 3 h and overnight using 4 mM H_2_O_2_ (*p* < 0.0001, *n* = 100) and elevated temperature at 37°C overnight (*p* < 0.03, *n* = 100) and 50°C for 2 h (*p* < 0.0001, *n* = 100), all showed increased DNA fragmentation (**Figure 1** and **Supplementary Figure S2**).

**Table 2:** Summary of the increased DNA damage and cell length after 2,4-D exposure.

DNA halo – long term exposure (μm)					
**3 h**			**15 h**		
**4 mM treated (*p* < 0.0001)**	**Formula**	**Control**	**4 mM treated (*p* < 0.0001)**	**Formula**	**Control**
4.4 ± 1.1	3.2 ± 0.8	3.1 ± 0.8	4.1 ± 0.9	3.3 ± 0.8	3.1 ± 0.6
91%^∗^			80%^∗^		
10%^∗∗^			10%^∗∗^		
0%^∗∗∗^			3%^∗∗∗^		

**DNA halo – short term exposure (μm)**					

**5 s (*p* < 0.03)**	**10 s (*p* < 0.0001)**	**30 s (*p* < 0.0001)**	**60 s (*p* < 0.0001)**	**Formula (60 s)**	**Control (60 s)**

1.85 ± 0.6	2.45 ± 0.8	2.47 ± 0.7	3.28 ± 1	1.54 ± 0.3	1.61 ± 0.3
50%^∗^	71%^∗^	60%^∗^	100%^∗^		
2%^∗∗^	16%^∗∗^	10%^∗∗^	41%^∗∗^		
0%^∗∗∗^	1%^∗∗∗^	3%^∗∗∗^	8%^∗∗∗^		

**Cell length (μm)**					

**3 h**			**15 h**		
**4 mM treated (*p* < 0.03)**	**Formula**	**Control**	**4 mM treated (*p* < 0.001)**	**Formula**	**Control**

2.05 ± 1.57	1.59 ± 0.50	1.69 ± 0.36	3.43 ± 2.85	1.34 ± 0.3	1.62 ± 0.30
78%^∗^			96%^∗^		
15%^∗∗^ 6%^∗∗∗^			28%^∗∗^ 15%^∗∗∗^		

*The numbers indicate average ± standard deviation, with percentage of cells as greater than average (^∗^), double the average (^∗∗^) and triple the average (^∗∗∗^) increase in DNA halo and cell length than that of the respective formula control.*

### Rapid Delocalization of FtsZ and FtsA in Dividing *E. coli*

*Escherichia coli* WM1074 (MG1655 lacU169) is a robust and fast growing strain (Archer et al., 2011), forming short rods visible during correlative imaging. Previous studies showed that 2,4-D at concentrations as low as 0.02 mM induces a filamentous phenotype and reactive oxygen species (Bhat et al., 2015b), indicating that 2,4-D impacts cell division in *E. coli*. In this study we exposed *E*. *coli* to 2,4-D while monitoring the localization of GFP-labeled cell division proteins FtsZ and FtsA in real-time using LSCM. Known ROS inducers, paraquat and hydrogen peroxide, were used as positive controls.

**FIGURE 2:**
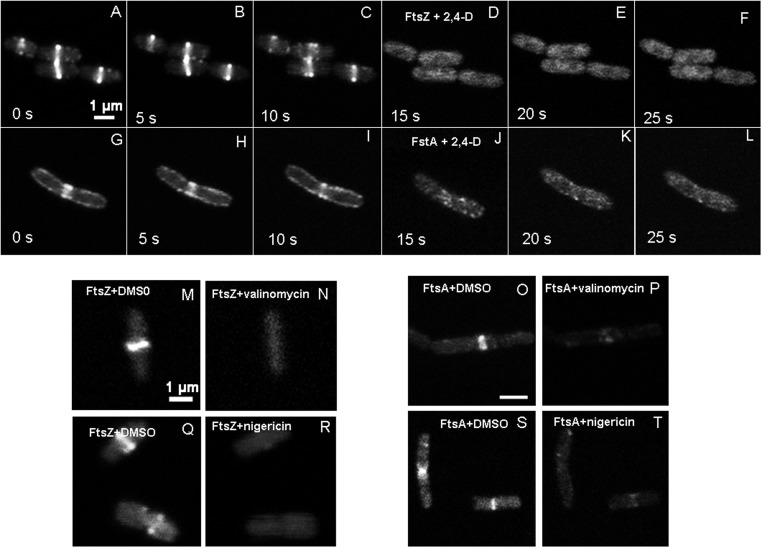
Time lapse images of live *E. coli* showing changes to FtsZ-GFP and GFP-FtsA localization imaged with LSCM (GFP 488/509 nm). In the absence of 2,4-D, *E. coli* showed FtsZ forming a centrally localized Z-ring **(A–C)**, but upon addition of 1 mM 2,4-D in the imaging medium, FtsZ dissociated from the Z-ring within 5 s forming bright spots **(D–F)**, mostly near the periphery and poles. GFP-FtsA also showed a similar change in localization **(G–L)**. The 1 mM 2,4-D was added to the imaging medium at 10 s. Valinomycin (30 μM) **(M–P)** and nigericin dissolved in DMSO (10 μM) **(Q–T)** also caused delocalization of FtsZ and FtsA, and DMSO control had no effects on FtsZ **(M,Q)** and FtsA **(O,S)**.

Time-lapse images taken in the absence of 2,4-D (sample control and formula control) show the presence of a distinct dynamic Z-ring at the mid-cell, partial rings and bright spots at sub-polar and polar regions (**Figures 2A–C**). Immediately following the addition of 1 mM 2,4-D, the Z-ring was perturbed within seconds, forming delocalized bright punctate fluorescence away from the center and toward cell periphery (**Figures 2D–F**). In the absence of 2,4-D, with <1 mM 2,4-D or formula treatment, GFP-FtsA localized in a manner similar to FtsZ-GFP, with a distinct ring structure at the mid-cell (**Figures 2G–I**). Immediately (<5 s) after the addition of 1 mM 2,4-D, GFP-FtsA delocalized and formed bright fluorescent masses (**Figures 2J–L**) more concentrated near the cell periphery. Relocalization of FtsZ-GFP and GFP-FtsA was observed for all cells exposed to 2,4-D (>100).

To determine whether the instant delocalization of FtsZ and FtsA could be induced by reactive oxygen species, we exposed *E. coli* FtsZ-GFP and GFP-FtsA to increasing concentrations of paraquat (0.01–20 mM) and hydrogen peroxide (0.01–10 mM), during live cell imaging in real time. Delocalization of FtsZ was not observed at any exposure level for either reagent (**Supplementary Figure S5**).

To determine the effects of membrane potential on the localization of FtsZ, and FtsA, *E. coli* was exposed to ionophores valinomycin and nigericin during real-time live cell imaging. At 30 μM valinomycin and at 10 μM nigericin there was complete delocalization of FtsZ and FtsA within 1 min (**Figures 2M–T**), resulting in diffuse fluorescence throughout the cell. Nigericin at 5 μM resulted in loss of the Z-ring structure in 50% of the cells, and at 10 μM the Z-ring was completely perturbed in the entire population within 1 min. DMSO had no effect on the localization of FtsZ and FtsA (**Figure 2**).

**FIGURE 3:**
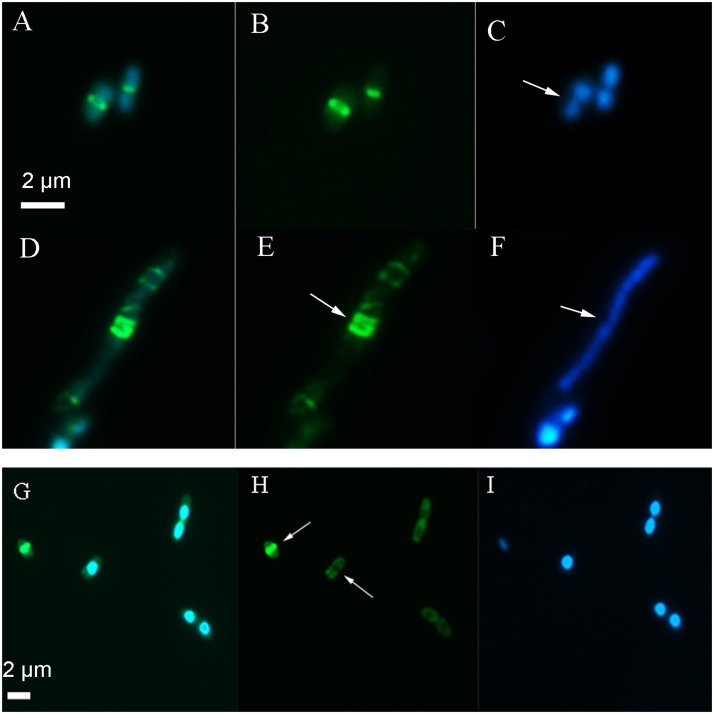
Images showing the location of the Z-ring and nucleoid after long term exposure to 2,4-D. In the absence of 2,4-D **(A–C)** *E. coli* showed a centrally localized Z-ring (**B**, arrow) with two nucleoids on either side (**C**, arrow). Exposure to 4 mM 2,4-D resulted in elongated cells with multiple partial Z-rings along the axis (**E**, arrow) and a large nucleoid mass (**F**, arrow) after an overnight exposure **(D–F)**. After 3 h exposure **(G–I)**, the majority of the cells lacked a typical Z-ring (**H**, arrow). The first panel shows the GFP+DAPI overlay, the second panel is only FtsZ-GFP and the third shows only the DAPI stain on the nucleoid.

Following several hours of live imaging in the presence of 2,4-D (**Supplementary Figure S3B**) and overnight (15 h) exposure to 2,4-D, *E. coli* showed a significantly elongated phenotype (*p* < 0.001, *n* ≥ 60), (**Figure 3**) compared to the formula and sample controls (*p* < 0.03, *n* ≥ 60) (**Table 2** and **Supplementary Figure S3**).

After 3 h exposure to 2,4-D, WM2026 (FtsZ-GFP) were stained with DAPI and imaged by epifluorescence to determine the location of the Z-ring in relation to the nucleoid, showing 70% of cells lacking a typical Z-ring (*n* ≥ 100) with a diffuse green fluorescence distributed throughout the cells but absent in nucleoid regions (**Figures 3G–I**). Elongated cells also showed large uneven nucleoid masses and multiple partially formed Z-rings along the axis (**Figures 3D–F**). Following overnight exposure (15 h) only 7% of the cells (*n* ≥ 100) had a typical Z-ring and a large number of cells showed irregular bright fluorescent spots mostly near the membrane. In the absence of 2,4-D, both the control and formula exposed cells had greater than 90% of cells with a centrally positioned Z-ring after 3 and 24 h.

### 2,4-D Dissipates Membrane Potential in *E. coli*

**FIGURE 4:**
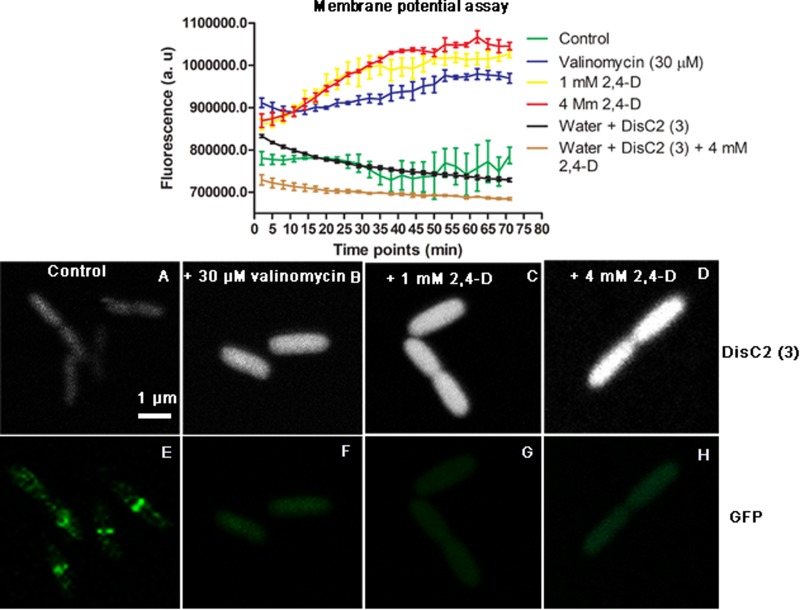
Membrane potential fluorescence assay with DisC2(3) during 2,4-D exposure. The plot shows a significant increase in fluorescence over time during exposure to 1 mM 2,4-D, 4 mM 2,4-D and the positive control valinomycin (30 μM) compared to the control. There is a slight reduction in fluorescence intensity over time with the dye in just water and 2,4-D. Representative images show *E. coli* with low fluorescence intensity and an intact Z-ring for controls **(A,E)** and increased fluorescence **(B–D)** accompanied by the absence of Z-rings **(F–H)** in the presence of 30 μM valinomycin, 1 mM 2,4-D and 4 mM 2,4-D.

After exposure to 1, 4 mM 2,4-D and 30 μM valinomycin, there was a significant increase in DisC2(3) fluorescence intensity over the course of 1 h (*p* < 0.0001), whereas unexposed *E. coli*, and wells containing water and dye or water, dye and 4 mM 2,4-D showed a slight decrease in fluorescence intensity (**Figure 4**). LSCM also showed cells having significantly increased DisC2(3) fluorescence signal in samples exposed to 2,4-D and valinomycin (**Figures 4A–D**).

### 2,4-D Alters the Intensity of SulAp-Gfp

There was no change to SulAp-GFP intensity immediately following exposure to 1 mM 2,4-D during live imaging. After an overnight exposure to 2,4-D, the SulAp-GFP intensity was significantly increased (*p* < 0.0001, *n* ≥ 100). Cells exposed to 2 and 4 mM 2,4-D showed a significantly (*p* < 0.0001) increased intensity of 303.2 ± 120.8 IU and 521.7 ± 159.0 IU, respectively, compared to those of the formula (137.1 ± 40.9 IU) and sample controls (154.3 ± 43.9 IU). The positive controls hydrogen peroxide (1 mM; 105.58 ± 29.3) and paraquat (1 mM; 102.9 ± 33.6 IU) also showed an increased SulAp-GFP intensity after overnight exposure compared to the control (38.3 ± 8.9 IU). In general elongated cells had a higher signal intensity compared to shorter cells (**Supplementary Figure S4**).

### 2,4-D Alters Surface Ultrastructure and Physical Properties

**FIGURE 5:**
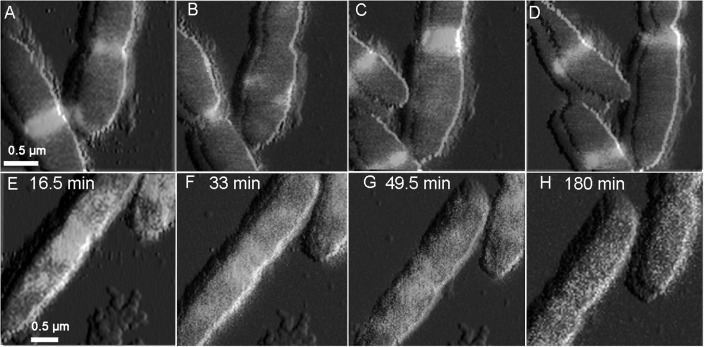
Overlaid time lapse images of simultaneously collected QI topography and LSCM images. In the absence of 2,4-D **(A–D)**, *E. coli* shows typical Z-ring localization at the mid-cell. Exposure to 4 mM 2,4-D **(E–H)** immediately halted cell constriction and caused disassembly of the Z-ring, with bright spotty fluorescence in the cytoplasm. The localization effects were not reversed after washing 2,4-D from the imaging medium **(H)**.

In the absence of 2,4-D, *E. coli* shows typical Z-ring localization at the mid-cell (**Figures 5A–D**), however after the addition of 4 mM 2,4-D, cell division arrested abruptly, regardless of the stage of cell division, and remained stagnant (**Figures 5E–H**). We removed 2,4-D and added fresh media after 4 h, but cell division did not resume even after washing (**Figure 4H**).

**FIGURE 6:**
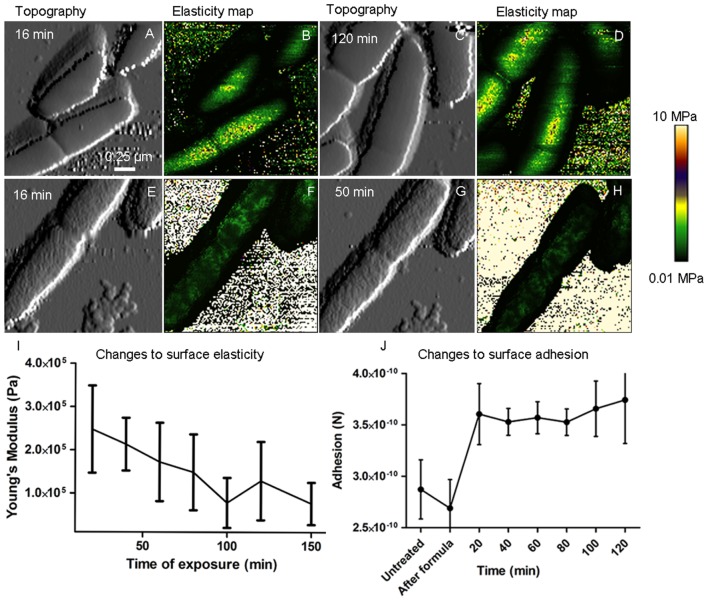
Time lapse AFM-QI^TM^ images show inhibition of cell division, changes to cell elasticity and changes to surface adhesion. QI^TM^ elasticity maps of the surface of live *E. coli* show no significant change in envelope elasticity during normal cell division **(B,D)**, however, there was a dramatic decrease in Young’s modulus after a 16 min exposure to 1 mM 2,4-D **(F)**, which further decreased after 50 min **(H)**. **(A,C,E,G)** Show corresponding topography. Plot **(I)** shows a decrease in elasticity as a function of time during 2,4-D exposure (*p* < 0.0001, *n* > 2000) and plot **(J)** shows a significant increase in adhesion (*p* < 0.0001, *n* > 2000) after 20 min, with no subsequent significant change.

QI^TM^ height images (**Figures 6A,C,E,G**) showed a significant change (*p* < 0.0001, *n* = 30) in surface roughness after a 20 min exposure to 4 mM 2,4-D, from 5.24 ± 3.23 nm (control) and 7.67 ± 2.23 (formula control) to 22.07 ± 12.2 nm, which did not change significantly after 20 min (*p* > 0.05). The high standard deviation of surface roughness following 2,4-D exposure indicates high variability. Young’s modulus, an estimate of cell envelope elasticity, was determined from QI^TM^ force curves at the center of the cell. Values were in the range of 1–4 MPa (**Figures 6B,D**) for cells in formula and prior to 2,4-D treatment, and did not change significantly during the course of cell division. Elasticity was significantly altered (*p* < 0.0001, *n* > 2000) with the addition of 4 mM 2,4-D, such that the average elasticity reduced to over a 100-fold after 20 min (**Figures 6F,H,I**) and was highly variable. In general, elasticity decreased as a function of exposure time, but to different degrees in each cell. Conversely, surface adhesion increased after 20 min 2,4-D exposure (*p* < 0.0001, *n* > 2000), but with no statistically significant increase thereafter (**Figure 6J**).

## Discussion

We have used correlative AFM- LSCM to reveal cell ultrastructural and mechanical changes concurrently with changes to the localization of cell division proteins in live cells in real time. Our results show that 2,4-D causes DNA damage immediately after the addition of 2,4-D, with increased damage during longer exposures. Altered FtsZ and FtsA localization within seconds, arresting cell division, was accompanied by depolarization of the cell membrane and increased SOS response. AFM showed simultaneous changes to surface physical properties during 2,4-D exposure in live *E. coli*. AFM-LSCM offers a molecular picture of the temporal dynamics of *E. coli* cellular division, providing mechanistic insights into the bacterial xenobiotic stress response mechanisms in real time.

### Rapid DNA Damage and SOS Induction

*Escherichia coli* WM1074 (Geissler et al., 2007) is a robust and fast growing environmental strain that serves as a good model to study the impact of xenobiotics. We previously demonstrated that 2,4-D produces ROS and induces a filamentous phenotype in *E. coli* BL21 DE3 and several genotypically diverse environmental strains at very low concentrations, implicating an impact on cell division (Bhat et al., 2015b). This herbicide also induced elongation in the strain under study (**Figure 3**) which was more pronounced after an overnight exposure. The rate of elongation was not consistent for all the cells, as some cells appeared more elongated than others, and there was a small population of cells that appeared to be unaffected, rod shaped with a distinct Z-ring (**Figures 3H**, **5A–D**). This discrepancy appears to be common, since individual cells react differently to external stress, including stress induced by mutation (Mileykovskaya et al., 1998; McCool et al., 2004; Justice et al., 2008; French et al., 2017). It is also interesting that compounds that directly interact and inhibit FtsZ produce a greater number of filamentous cells, nonetheless they produce cells with variable lengths (Araujo-Bazan et al., 2016). It is to be expected that not all cells in a given population are in the same metabolic state. If we consider for example stress associated with DNA damage, the cell has a number of different ways to respond (Uphoff et al., 2013) which will directly impact the level of stress experienced by the cell. Furthermore, every molecular process in the cell is in a constant state of flux, with only a snap shot observed during the imaging process. Consistent with this idea, 6 and 15% of the cells are filamentous after 3 and 15 h, respectively, greater than three times that of the control. The filamentous cells, as expected, showed increased DNA fragmentation and SOS response, indicating that the elongated cells were under greater stress compared to average sized cells (**Supplementary Figure S4**).

There was no immediate increase in SulAp-GFP during 2,4-D exposure, likely since any subtle immediate effects would be below the detection limit of the microscope and could be convoluted with photo-bleaching. The increased SulAp-GFP intensity after 3 h and overnight exposure indicates increased SOS response due to oxidative stress. Oxidative stress that induces DNA damage in bacteria activates RecA, a coprotease that helps autocleave the LexA repressor. In the absence of LexA, the operator sequences allow expression of more than 40 SOS genes, including SulA, which halt cell division and repair damaged DNA (Janion, 2008). *E. coli* WM2739 is a strain with SulAp-GFP, a reporter of the SOS response with the SulA promoter under the control of LexA regulation. SulAp-GFP showed a uniformly dim signal in the absence of 2,4-D, indicating low constitutive basal SulA expression, and an increase in expression indicative of oxidative stress and DNA damage (McCool et al., 2004; Nazir and Harinarayanan, 2015). SulA is a major repair protein in the SOS operon which inhibits cell division, prolonging the cell cycle during which the damaged DNA can be repaired (Imlay, 2013). An increase in the SulA:FtsZ ratio is known to cause cell filamentation, since SulA binds to FtsZ when present at higher concentrations, inhibiting cell division (Dajkovic et al., 2008). SulA works by interacting with the catalytic site on FtsZ, helping to disassemble the existing Z-ring and sequestering FtsZ monomers to prevent its further assembly (Huang et al., 1996; Chen et al., 2012), all consistent with the filamentous phenotype observed at longer exposure times (**Figure 3**). Elongated cells also produced more SulAp-GFP compared to average sized cells, lending further support for this idea (**Supplementary Figure S4**).

### Implications of 2,4-D and Changes to Membrane Potential on Z-Ring Assembly

Cell division in *E. coli* is initiated by septum formation, facilitated by the accumulation of FtsZ and its colocalizing partners, which form a membrane associated complex called the divisome (Natale et al., 2013). FtsZ undergoes GTP-dependent self-polymerization to form a highly dynamic scaffold called the Z-ring, for which division proteins are in constant flux between the cytosol and the divisome (Anderson et al., 2004). FtsZ-GFP was observed as bright fluorescent spots that moved along the cell’s longitudinal axis and formed mid-cell bands that eventually constrict, all the while forming a second ring at the middle of daughter cells preparing for the next division (Adams and Errington, 2009). Cell constriction was clearly visible in the AFM images, for which constriction began following complete Z-ring formation observed by LSCM (**Figure 4**).

We monitored the effects of 2,4-D exposure by fluorescence assay using the carbocyanine dye, 3,3^′^-diethylthiacarbocyanine iodide [DisC2(3)], known to be a good indicator of membrane potential (Epand et al., 2010; Te Winkel et al., 2016). *E. coli* membrane potential began to dissipate almost immediately following exposure (5 min) to 1 mM, 4 mM 2,4-D and 30 μM valinomycin, as indicated by a significantly increased fluorescence signal (**Figure 4**). There was reduced fluorescence in control cells, consistent with fluorescence quenching in polarized cells. A reduction in florescence with just water and 2,4-D indicates a possible reaction between the two, but regardless, treated samples had significantly increased fluorescence (*p* < 0.0001), indicating a rapid loss in membrane potential. It is well known that 2,4-D is lipophilic, altering the fluidity of the cell membrane (Fabra de Peretti et al., 1992; Viegas et al., 2005), and affecting oxidative phosphorylation (Bhat et al., 2015b). This is the first study to show 2,4-D directly causing membrane depolarization, resulting in an immediate loss of the Z-ring and cell division.

FtsZ-GFP and DisC2(3) were imaged simultaneously by LSCM to confirm the loss of the Z-ring, along with the dissipation of the membrane potential. Indeed, cells exposed to 1 mM, 4 mM 2,4-D and valinomycin had no Z-rings and increased fluorescence, the latter indicating depolarized membranes (**Figures 4A–H**). There was also variation in the DisC2 intensity in different cells, showing heterogeneity of the stress response.

The immediate change in FtsZ localization (**Figure 2**) when exposed to 1 mM 2,4-D, valinomycin and nigericin can be largely explained by changes to membrane potential. The positive control valinomycin is a K^+^ carrier ionophore that specifically dissipates the membrane potential. Nigericin facilitates electroneutral exchange of H^+^ and K^+^ ions, thereby depleting ΔpH between the cytosol and external environment (Ahmed and Booth, 1983). Rapid perturbation of the Z-ring during 2,4-D exposure in a manner similar to those of the positive controls indicates that 2,4-D disassembles the divisome complex through alteration to the membrane potential. Absence of the Z-ring during longer 2,4-D exposures indicates the cell’s inability to reverse divisome disassembly with increased DNA damage. Sub-lethal 2,4-D (1 mM) is insufficient to kill all cells in the population, some of which likely overcome the stress and continue to divide, consistent with some *E. coli* showing Z-rings following 2,4-D exposure after the 3 h mark (**Figure 3H**).

Our previous metabolomics study showed that 2,4-D inhibits oxidative phosphorylation in *E. coli* (Bhat et al., 2015b) known to be a direct consequence of membrane potential dissipation (Lewis et al., 1994). Since FtsZ is GTP dependent, loss of FtsZ assembly may be a direct consequence of changes in cellular respiration and membrane potential. Membrane potential is crucial for the stability of the divisome complex (Strahl and Hamoen, 2010), which could directly result in Z-ring collapse on the time scales observed in this study. Indole, a structural analog of 2,4-D, blocks *E. coli* cell division by disrupting the MinCD oscillation as a result of reduced membrane potential crucial for cell division (Chimerel et al., 2012). Consistent with this idea, we observed FtsA changing its localization in a manner similar to FtsZ following 2,4-D exposure (**Figures 2G–L**). It has been demonstrated that FtsA and ZipA compete for the C-terminal end of FtsZ to form membrane tethers and colocalize within the Z-ring structure. It has been shown that upon SulA induction and disassembly of the Z-ring, FtsZ, FtsA and ZipA appear as punctate, short polymeric structures, appearing as patches on the membrane (Rowlett and Margolin, 2014). Altered membrane organization induced by 2,4-D would have a direct impact on membrane-associated FtsA, which could lead to disassembly of the Z-ring. Changes to FtsA localization and its patchy distribution indicate that 2,4-D may alter the assembly of the entire divisome, possibly impacting other cell division proteins.

Previous studies show 2,4-D to bind directly and affect polymerization of purified neuronal tubulin *in vitro* (Rosso et al., 2000), so it could have a similar impact on its structural homolog FtsZ in *E. coli.* Changes to FtsZ structures have been previously documented in *E. coli*, showing punctate polymeric structures throughout the cell upon its disassembly, with the induction of SulA (Rowlett and Margolin, 2014; Vedyaykin et al., 2014). FtsZ in *Bacillus subtilis* exposed to benzamide appears as randomly distributed dynamic foci, having no specific localization (Adams et al., 2011). Resveratrol inhibits *E. coli* division and causes elongation by inhibiting Z-ring formation and FtsZ expression, causing DNA damage and an upregulated SOS response (Hwang and Lim, 2015). Together with this study, it can be concluded that 2,4-D disrupts the divisome by immediately dissipating the membrane potential, causes DNA damage which induces the SOS response, ultimately leading to cell elongation during longer exposures.

### Changes to Roughness, Elasticity and Adhesion in Live Cells during 2,4-D Exposure

Although no prior study has characterized the effects of aromatic compounds on the bacterial surface in live cells, there are extensive studies on fixed cells imaged in air following exposure to antimicrobial peptides (Fantner et al., 2010), essential oils (La Storia et al., 2011) and antibiotics (Yang et al., 2006), all showing changes to surface ultrastructure as a primary response to external stress. Our results are consistent with the impact of other antimicrobial compounds, with alterations to surface roughness and envelope elasticity, but we have resolved the temporal changes. *E. coli* exposed to sub-lethal levels of 2,4-D had a 100-fold lower surface elasticity within 30 min, further reduced over a period of a few hours, indicating changes to envelope compliance over time. Removal of 2,4-D from the imaging medium did not reverse this effect, indicating an irreversible alteration to cell envelope physical properties and implying that 2,4-D likely perturbs a select group of cell surface molecules.

The outer membrane of *E. coli* is highly complex and asymmetric, made up of lipids, long chain lipopolysaccharides (LPS) and membrane proteins (Kleanthous and Armitage, 2015). Known to be lipophilic (Benndorf et al., 2006), 2,4-D likely reacts with LPS which could lead to the observed changes in surface elasticity and adhesion. The *E. coli* cell surface contains ∼90% LPS, making its surface negatively charged, consistent with lower adhesion between the negatively charged silicon tip and bacteria compared to the Cell-Tak^TM^ covered glass surface. We speculate that an increase in adhesion immediately after exposure to 2,4-D (**Figure 6J**) likely indicates a rearrangement of LPS, in accordance with more compliant and rougher cells. We did not observe increased adhesion after 20 min, indicating that 2,4-D irreversibly interacts in a short period of time, likely with the entire surface exposed LPS.

This is the first report showing that 2,4-D is capable of arresting cell division in *E. coli* within seconds by altering membrane potential, FtsZ and FtsA localization, accompanied by DNA damage and the SOS response. Longer exposures resulted in an even greater SOS response, irregular Z-ring formation and improperly divided cells, giving rise to cell filamentation. Simultaneous real-time AFM imaging of live *E. coli* showed time-dependent changes to surface roughness, adhesion and elasticity, indicating a direct interaction between 2,4-D and the envelope surface.

## Author Contributions

SB, BK, AK, and ZS designed the experiments, analyzed the data, and wrote the paper. SB and TD designed the experiments, and wrote and revised the manuscript. All authors gave final approval for publication.

## Conflict of Interest Statement

The authors declare that the research was conducted in the absence of any commercial or financial relationships that could be construed as a potential conflict of interest.

## Supplementary Material

FIGURE S1 *Escherichia coli* growth curve at different 2,4-D exposure concentrations. The formula sample contains all the ingredients in the formulation without any 2,4-D, see methods for more details on components of the formulation.

FIGURE S2 Epifluorescence images (SYBR gold 497/537 nm) showing increased DNA damage during long exposure to 2,4-D. Images show increased DNA spreading in *E. coli* exposed to 4 mM 2,4-D **(C,F)** after 3 h **(C)**, 15 h **(F)** compared to the corresponding formula exposed **(B,E)** and control cells **(A,D)**. Cells exposed to 4 mM H_2_O_2_ for 3 h **(H)** and 15 h **(I)** and increased temperature at 37°C overnight **(K)** and 50°C for 2 h **(L)** also had significantly larger halos (*p* < 0.0001, *n* = 100) compared to their representative controls without H_2_O_2_ **(G)** and grown at 30°C **(J)**, respectively.

FIGURE S3 Scatter plot showing the increase in cell length after 2,4-D exposure. *E. coli* showed an increased cell length after 3 h and 15 h exposure to 4 mM 2,4-D, compared to the formula control (FC) and control cells (C). The samples exposed for 3 h had fewer filamentous cells than those exposed for 15 h. **(A,B)** Show representative DIC images of control and 2,4-D treated cells.

FIGURE S4 Changes to SulAp-GFP intensity after 3 h and overnight exposure to 4 mM 2,4-D imaged using epifluorescence microscopy (GFP 488/509 nm). Formula treated *E. coli* **(A)** show a uniformly low signal from SulA-GFP, however, after 3 h exposure to 4 mM 2,4-D **(B)** cells show an overall brighter signal **(C)** and elongated cells have a more intense signal compared to the shorter cells after overnight exposure. The positive controls, paraquat **(D–F)** and hydrogen peroxide **(G–I)**, also show an increased SulAp-GFP intensity after overnight exposure compared to their respective controls.

FIGURE S5 LSCM images of *E. coli* showing no change in localization of FtsZ-GFP and GFP-FtsA after exposure to different concentrations of paraquat and hydrogen peroxide.
