# Supplementary figures and images for: Exposure to Sub-lethal 2,4-Dichlorophenoxyacetic Acid Arrests Cell Division and Alters Cell Surface Properties in Escherichia coli
Source: Front Microbiol. 2018 Feb 1;9:44. doi: 10.3389/fmicb.2018.00044 (PMC5810288; doi:10.3389/fmicb.2018.00044)

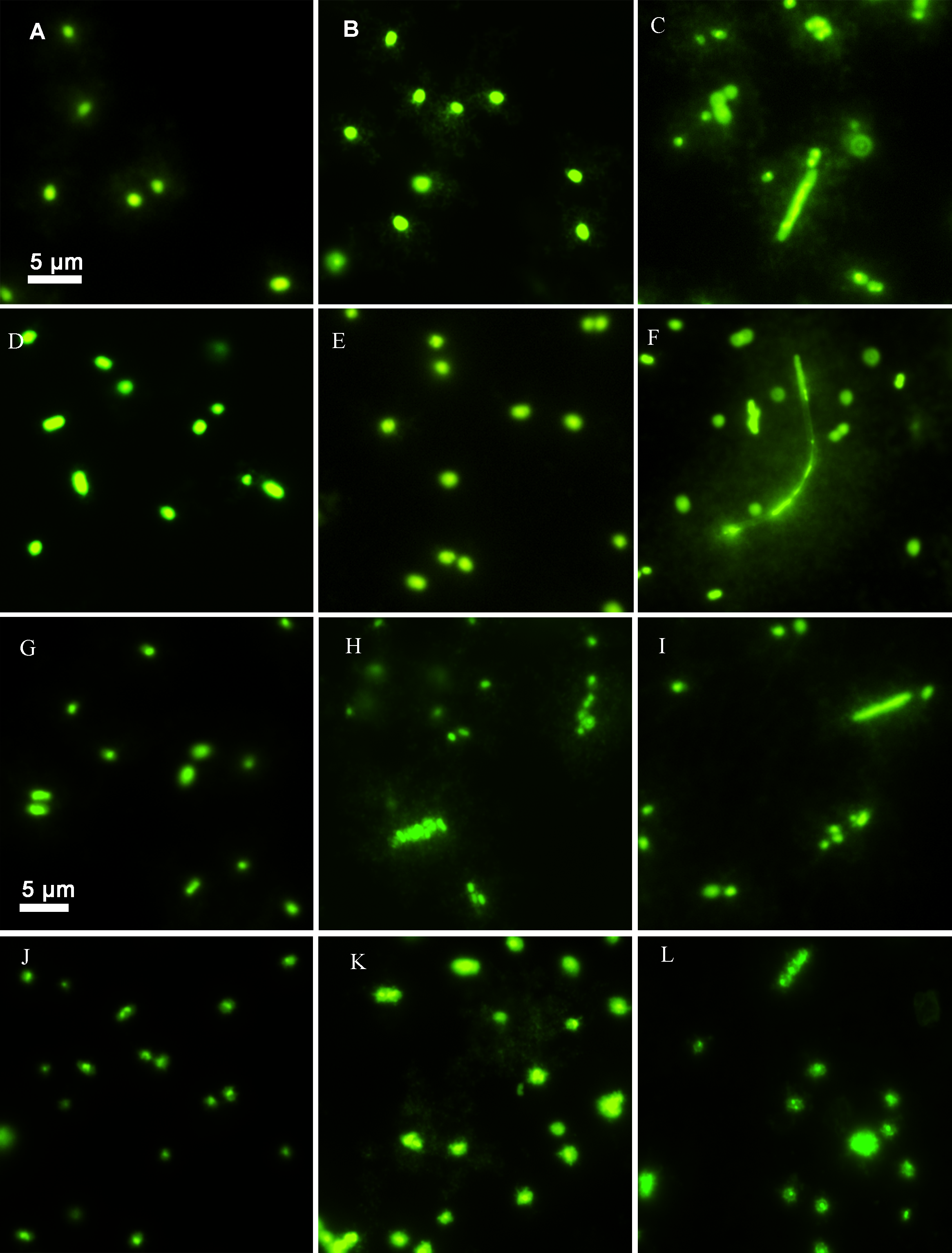

Supplement: FIGURE S2 — Epifluorescence images (SYBR gold 497/537 nm) showing increased DNA damage during long exposure to 2,4-D. Images show increased DNA spreading in E. coli exposed to 4 mM 2,4-D (C,F) after 3 h (C), 15 h (F) compared to the corresponding formula exposed (B,E) and control cells (A,D). Cells exposed to 4 mM H2O2 for 3 h (H) and 15 h (I) and increased temperature at 37°C overnight (K) and 50°C for 2 h (L) also had significantly larger halos (p < 0.0001, n = 100) compared to their representative controls without H2O2 (G) and grown at 30°C (J), respectively. [file Image_2.TIF]

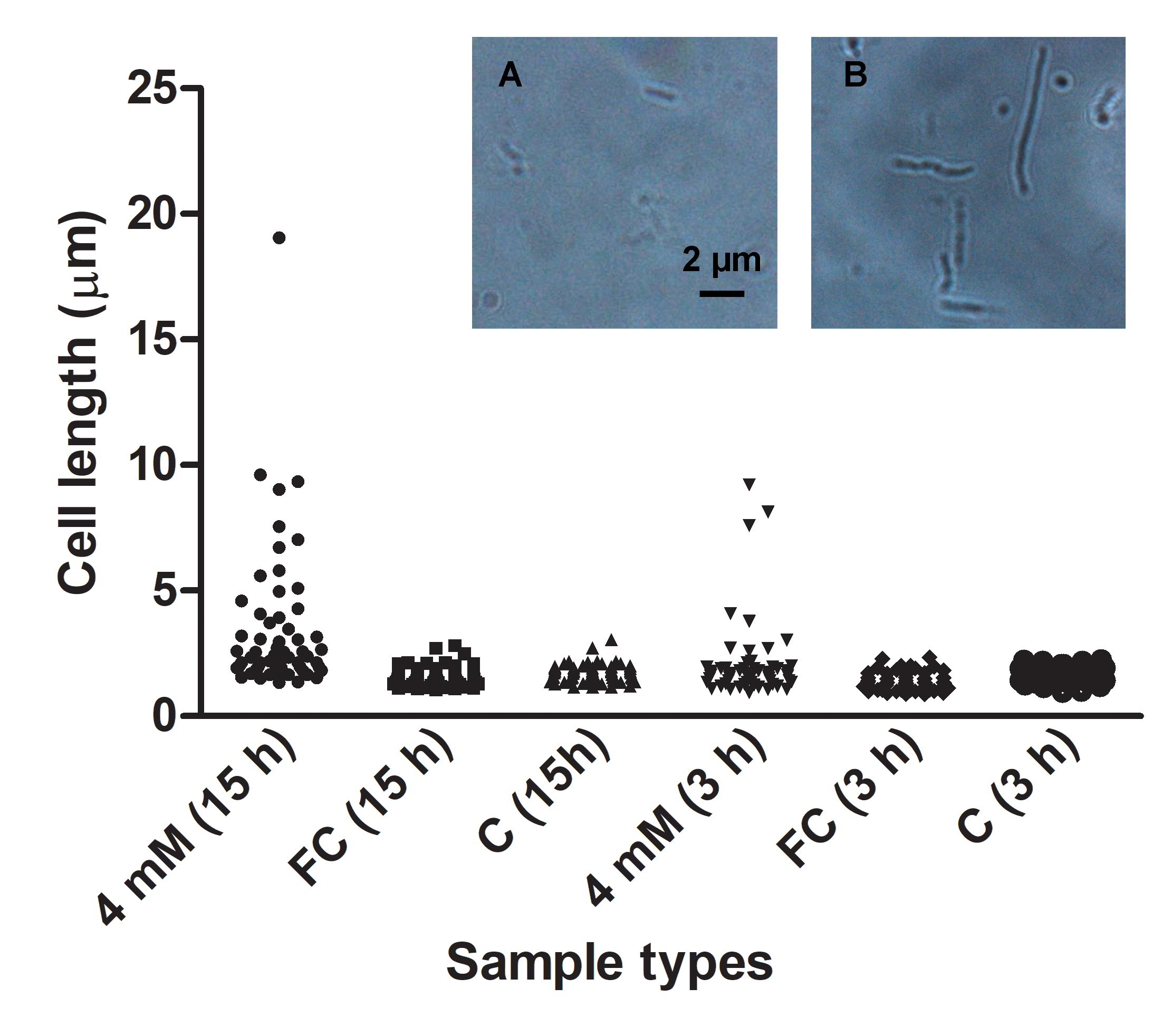

Supplement: FIGURE S3 — Scatter plot showing the increase in cell length after 2,4-D exposure. E. coli showed an increased cell length after 3 h and 15 h exposure to 4 mM 2,4-D, compared to the formula control (FC) and control cells (C). The samples exposed for 3 h had fewer filamentous cells than those exposed for 15 h. (A,B) Show representative DIC images of control and 2,4-D treated cells. [file Image_3.TIF]

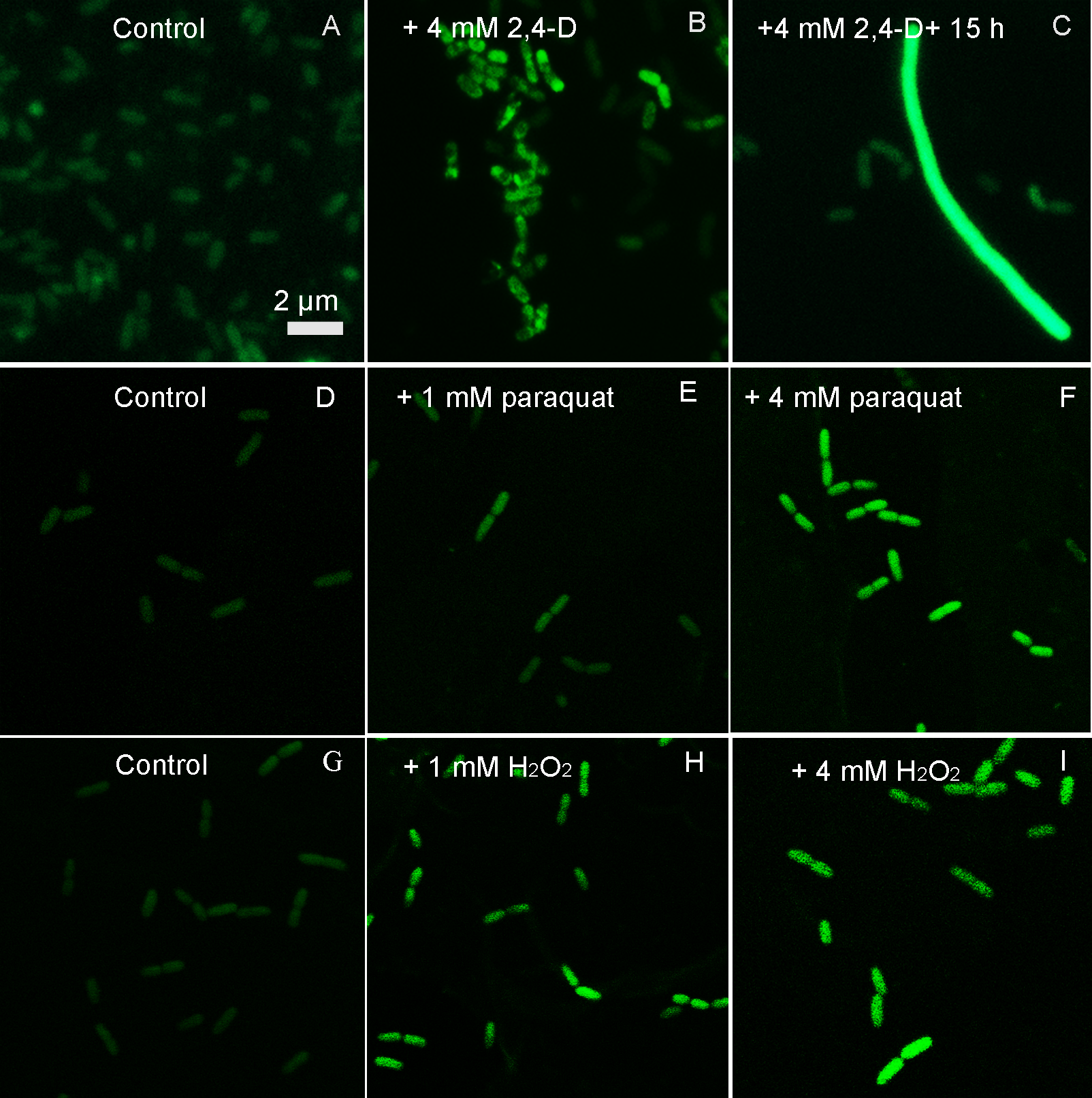

Supplement: FIGURE S4 — Changes to SulAp-GFP intensity after 3 h and overnight exposure to 4 mM 2,4-D imaged using epifluorescence microscopy (GFP 488/509 nm). Formula treated E. coli (A) show a uniformly low signal from SulA-GFP, however, after 3 h exposure to 4 mM 2,4-D (B) cells show an overall brighter signal (C) and elongated cells have a more intense signal compared to the shorter cells after overnight exposure. The positive controls, paraquat (D–F) and hydrogen peroxide (G–I), also show an increased SulAp-GFP intensity after overnight exposure compared to their respective controls. [file Image_4.TIF]

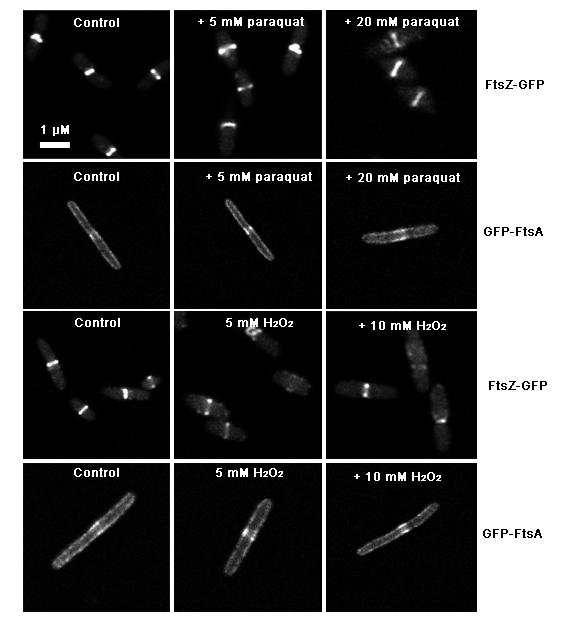

Supplement: FIGURE S5 — LSCM images of E. coli showing no change in localization of FtsZ-GFP and GFP-FtsA after exposure to different concentrations of paraquat and hydrogen peroxide. [file Image_5.TIF]
